# Integrating genotype–phenotype correlations into the diagnostic workflow of odontogenic tumors and cysts: insights from a 69 lesion cohort

**DOI:** 10.1007/s00784-025-06642-5

**Published:** 2025-12-09

**Authors:** Sibel Elif Gültekin, Burcu Toközlü, Carina Heydt, Reinhard Büttner

**Affiliations:** 1 Faculty of Dentistry, Department of Oral Pathology, Gazi University, Ankara, 06510 Turkey; 2 Faculty of Medicine, Institute of Pathology, University of Cologne, Cologne, Germany

**Keywords:** Odontogenic tumors, Odontogenic cysts, Next-generation sequencing, Somatic mutations, Molecular profiling, Diagnostic pathology

## Abstract

**Objectives:**

Odontogenic tumors (OTs) represent a heterogeneous group of lesions ranging from hamartomatous or non-neoplastic proliferations to benign and malignant neoplasms with metastatic potential. Their classification remains challenging due to morphological and molecular variability. In this study, we performed comprehensive molecular profiling of odontogenic lesions—excluding previously analyzed ameloblastomas—to identify genomic alterations that define molecular subtypes and evaluate their diagnostic significance and potential therapeutic implications.

**Materials and methods:**

A total of 88 odontogenic lesions were analyzed, including 25 cysts and 63 benign or malignant tumors, classified according to the 2022 5th edition of the World Health Organization (WHO) classification. Targeted next-generation sequencing (NGS) was performed to detect somatic mutations. Mutational patterns were correlated with histological subtypes and clinical parameters such as age, gender, lesion location, and recurrence.

**Results:**

Somatic mutations were identified in 46 of 69 lesions successfully sequenced (66.7%). Among malignant tumors, genomic alterations—including gene fusions and mutations—were detected in 75% of cases. The highest mutation frequency was observed in OTs of epithelial origin. Notably, 11 of the 46 mutated cases harbored concurrent alterations in two genes.

**Conclusions:**

Molecular profiling serves as a valuable diagnostic adjunct in evaluating odontogenic tumors and cysts. Incorporating mutational analysis into routine workflows may enhance diagnostic precision, particularly in small or ambiguous biopsies, and may provide preliminary insights for future development of targeted therapeutic approaches.

**Clinical relevance:**

This study highlights the diagnostic value of integrating somatic mutation analysis into the standard evaluation of odontogenic lesions and highlights its potential adjunctive role in diagnostically challenging cases and as a basis for future therapeutic investigations.

## Introduction

Odontogenic tumors (OTs) and cysts represent a diverse and complex group of lesions derived from the odontogenic epithelium, ectomesenchyme, or both. These lesions range from hamartomatous proliferations to aggressive neoplasms with significant recurrence potential and, in rare instances, metastatic behavior [1]. Their classification remains challenging due to overlapping clinical, radiological, and histopathological features, as well as their capacity for phenotypic variability and progression. While the World Health Organization (WHO) 2022 classification of odontogenic lesions builds upon earlier frameworks, it remains primarily morphology-based and does not fully account for molecular alterations that may underlie tumor behavior and biological aggressiveness [2, 3].

Although the majority of odontogenic tumors are benign, treatment decisions—ranging from conservative curettage to radical resection—can significantly impact patient morbidity, especially in younger individuals. Accurate diagnosis is therefore crucial but often hampered by small incisional biopsies, cystic architecture, or histologic mimicry between lesions. For instance, ameloblastoma-like epithelium can be seen in a variety of odontogenic entities, and the diagnostic process may be further complicated when mucous cells, keratinization, or ghost cells are present. In routine histopathological practice, these challenges are exacerbated by the limited tissue available from incisional biopsies, which are frequently obtained from the most accessible yet histologically ambiguous parts of the lesion [4–6].

Recent advances have identified recurrent alterations in MAPK and Hedgehog pathways [3, 7–9]. In particular, BRAF p.V600E is strongly associated with ameloblastomas, while PTCH1 mutations predominate in odontogenic keratocysts. These molecular findings provide new perspectives on the pathogenesis and potential targeted treatment of odontogenic lesions. Similarly, SMO and PTCH1 mutations have been implicated in keratocystic and other odontogenic lesions, providing novel diagnostic and therapeutic insights [8, 10–13]. Next-generation sequencing (NGS) and related molecular profiling technologies offer the potential to complement histopathological evaluation by uncovering genotype–phenotype correlations that may assist in classifying diagnostically ambiguous cases and guiding individualized therapy [14, 15].

In light of these developments [9–14], integrating genetic analysis into routine diagnostic workflows may enhance diagnostic accuracy and provide actionable information, especially in limited biopsy samples where histologic resolution is insufficient. Despite this promise, there is still a paucity of studies systematically correlating molecular findings with clinicopathologic features across a broad range of odontogenic lesions.

The present study aims to address this gap by performing targeted NGS-based molecular profiling in a series of 69 odontogenic tumors and cysts collected from referral centers in Turkey and Germany. We evaluated the association of genetic alterations with histopathological diagnosis and clinical features, with particular attention to the diagnostic utility of genotyping in small biopsies. This approach may contribute to more accurate classification, guide surgical management, and inform future development of targeted therapies.

## Materials and methods

### Tumor specimens, histological and clinical data

A total of 88 odontogenic lesions were retrospectively retrieved from the archives of the Department of Oral Pathology, Faculty of Dentistry, Gazi University (Ankara, Turkey) and the Institute of Pathology, Cologne University Medical Faculty (Cologne, Germany). The cohort included 63 odontogenic tumors (OTs) and 25 odontogenic cysts (OCs). Ethics approvals were obtained from both institutions (University of Cologne: No. 13–091; Gazi University: No. 77082166-604.01.01.01.02–2017−03).

All cases were reviewed and reclassified by two experienced oral pathologists (SEG, BT) using hematoxylin and eosin (H&E) stained slides, according to the 2022 WHO classification of Head and Neck Tumors (Table 1) [2]. Histological grading of dysplasia was performed independently by two pathologists. Interobserver agreement was high (κ = 0.89), indicating excellent concordance.

Lesions were categorized as follows:

- Benign epithelial odontogenic tumors (*n* = 16): adenomatoid odontogenic tumor (AOT), squamous odontogenic tumor (SOT), calcifying epithelial odontogenic tumor (CEOT).
- Benign mixed epithelial and mesenchymal tumors (*n* = 31): ameloblastic fibroma (AF), odontoma, dentinogenic ghost cell tumor.
- Benign mesenchymal odontogenic tumors (*n* = 12): odontogenic fibroma (OF) including hybrid tumors, odontogenic myxoma (OM).
- Malignant odontogenic tumors (*n* = 4): clear cell odontogenic carcinoma (CCOC), odontogenic sarcoma (OS).
- Odontogenic cysts (*n* = 25): calcifying odontogenic cysts (COC, *n* = 15), odontogenic keratocysts (OKC, *n* = 10) (Table 2) [2, 3].

**Table 1:** Odontogenic cysts and tumors according to 2022 WHO classification [2]

Odontogenic Tumours
**Benign epithelial odontogenic tumours** Adenomatoid odontogenic tumour Squamous odontogenic tumour Calcifying epithelial odontogenic tumour Ameloblastoma, unicystic Ameloblastoma, extraosseous/peripheral Ameloblastoma, conventional Adenoid ameloblastoma Metastasizing ameloblastoma
Benign mixed epithelial & mesenchymal odontogenic tumours Odontoma Primordial odontogenic tumour Ameloblastic fibroma Dentinogenic ghost cell tumour
***Benign mesenchymal odontogenic tumours*** Odontogenic fibroma Cementoblastoma Cemento-ossifying fibroma Odontogenic myxoma
***Malignant odontogenic tumours*** Sclerosing odontogenic carcinoma Ameloblastic carcinoma Clear cell odontogenic carcinoma Ghost cell odontogenic carcinoma Primary intraosseous carcinoma, NOS Odontogenic carcinosarcoma Odontogenic sarcomas
**Odontogenic Cysts**
Radicular cyst Inflammatory collateral cysts Surgical ciliated cyst Nasopalatine duct cyst Gingival cysts Dentigerous cyst Orthokeratinised odontogenic cyst Lateral periodontal cyst and botryoid odontogenic cyst Calcifying odontogenic cyst Glandular odontogenic cyst Odontogenic keratocyst

**Table 2:** Demographic data, histopathological classification and distribution of odontogenic lesions

	Age	Gender, *n* (%)		Location, *n* (%)		Total
	Mean	F	M	Mandible	Maxilla	*n* (%)
Benign Tumors						**59 (60.2)**
Epithelial Origin						**16 (18**,**1)**
Adenomatoid Odontogenic Tumor	23.78	11 (12.5)	3 (0.3)	9 (10)	5 (0.56)	14 (15.9)
Squamous Odontogenic Tumor	56	0	1 (0.11)	1 (0.11)	0	1 (1.1)
CEOT (Pindborg tumor)	44	0	1 (0.11)	1 (0.11)	0	1 (1.1)
Mixed Origin						**31 (34**,**1)**
Ameloblastic Fibroma	12	5(0.56)	1 (0.11)	4 (0.45)	2 (0.22)	6 (6.8)
Odontoma	15.33	9 (10)	15 (17)	13 (14)	11 (12.5)	24 (27.3)
Mesenchymal Origin						**12 (14**,**7)**
OdontogenicFibroma	24.5	2(0.22)	2 (0.22)	2 (0.22)	2 (0.22)	4 (4.5)
Myxoma & Fibromyxoma	33.37	6 (0.68)	2 (0.22)	7 (0.8)	1 (0.11)	8 (9.1)
Dentinogenic Ghost Cell Tumor	14	0	1 (0.11)	1(0.11)	0	1 (1.1)
Malignant Tumors						**4 (4.5)**
Odontogenic Clear Cell Carcinoma	61.66	1(0.11)	2 (0.22)	2 (0.22)	1 (0.11)	3 (3.4)
Odontogenic Sarcoma	14	0	1 (0.11)	1 (0.11)	0	1 (1.1)
Odontogenic Cysts						**25 (28.4)**
Calcifying Odontogenic Cyst	45.66	7 (0.8)	8 (0.90)	14 (15)	1 (0.11)	15 (17.0)
Odontogenic Keratocyst	37	7 (0.8)	3 (0.3)	10 (11)	0	10 (11.4)
TOTAL	**27**,**5 ± 19.5**	**48 (54**,**5)**	**40 (45**,**5)**	**65**	**23**	**88 (100)**

## Tissue preparation and molecular profiling

All samples were formalin-fixed, paraffin-embedded (FFPE), and in some cases decalcified according to standard protocols. From each block, three to six 10-µm thick unstained sections were cut for DNA extraction. Tumor-rich areas were identified on matching H&E-stained slides and manually microdissected.

**Fig. 1:**
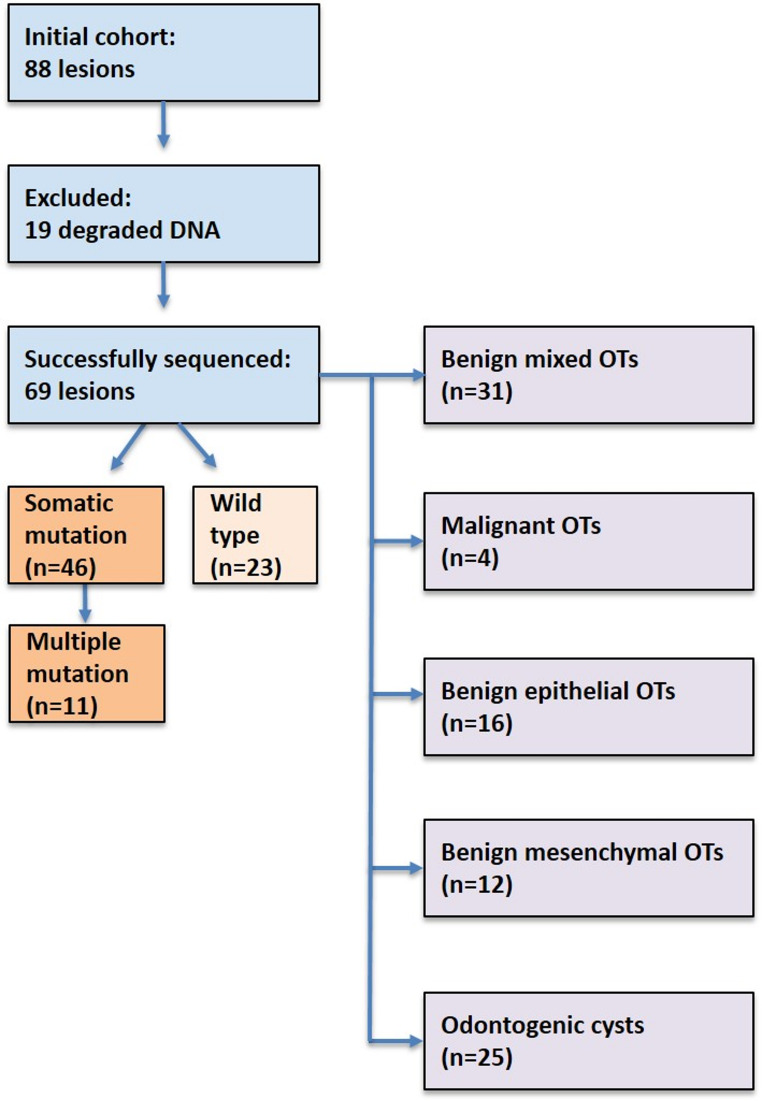
Flowchart of the study cohort. Of 88 odontogenic lesions, 69 were successfully sequenced after DNA quality control. Cases were stratified by mutation status and classified according to the 2022 WHO [2] scheme: benign mixed, epithelial, mesenchymal, malignant tumors, and odontogenic cysts

DNA extraction was performed using the Maxwell^®^ 16 FFPE Plus Tissue LEV DNA Purification Kit (Promega). DNA quality and concentration were assessed by qPCR. High-quality DNA suitable for next-generation sequencing (NGS) was obtained from 69 of the 88 lesions; the remaining 19 samples were excluded due to degraded DNA, likely resulting from harsh decalcification or block aging (Fig. 1) [8].

NGS was performed using customized gene panel covering 28 oncogenes and tumor suppressors relevant to odontogenic tumorigenesis (*ARAF*,* BRAF*,* CDK4*,* CDKN2A*,* CTNNB1*,* DDR2*,* EGFR*,* ERBB2*,* FGFR2*,* FGFR3*,* GNA11*,* GNAQ*,* HRAS*,* IDH1*,* KEAP1*,* KIT*,* KNSTRN*,* KRAS*,* MAP2K1*,* MET*,* NFE2L2*,* NRAS*,* OXA1L*,* PDGFRA*,* PIK3CA*,* PTEN*,* RAC1*). In addition, PTCH1 and FGFR1 were included to ensure coverage of SHH and FGF pathway alterations. Library preparation was performed using either Qiagen GeneRead DNAseq Panels V2 or Twist Custom Panels, followed by sequencing on Illumina MiSeq or NextSeq platforms.

The mutation calling threshold was set at:

- Allele frequency (AF) ≥ 5%.
- Coverage depth (COV) ≥ 200×.

In selected cases of clear cell odontogenic carcinoma (CCOC), fluorescence in situ hybridization (FISH) analysis was performed using a dual-color break-apart probe for *EWSR1* (ZytoVision, Germany) to detect chromosomal translocations involving chromosome 22q12.

## Clinical data and statistical analysis

Clinical and histopathological variables (age, gender, location, recurrence, tumor type) were analyzed according to mutation status. Categorical data were expressed as frequency (percentage), and associations were evaluated using the Chi-square test.

To assess correlation between molecular findings and clinical parameters, Spearman’s rank correlation test was applied. All statistical analyses were performed using SPSS software version 22.0 (IBM Corp., Armonk, NY, USA). A *p*-value < 0.05 was considered statistically significant.

## Results

### Demographic and clinical characteristics

The demographic and clinical distribution of the lesions is summarized in Table 1. At the time of diagnosis, the mean age of patients was 27.5 ± 19.5 years (range: 1–79 years). Patients with benign OTs were significantly younger than those with malignant tumors (*p* = 0.043). The youngest subgroup was AF with a mean age of 12 years, while the oldest patients were seen in the CCOC group (mean age: 61.6 years).

The gender distribution was nearly balanced (45.5% male, 54.5% female). The mandibular posterior region was the most frequent location across all lesion types. Almost all odontogenic cysts (except one) were located in the mandible, a statistically significant difference compared to tumors (*p* = 0.004).

### Mutational landscape and histopathologic correlation

Association between mutation status and clinicopathological variables of patients with odontogenic lesions is given in Table 3.

**Table 3:** Association between mutation status and clinicopathological variables of patients with odontogenic lesions

	Mutation Status		
Variables	**Wild Type**, ***n*** **(%)**	**Mutation**, ***n*** **(%)**	***P***
Age (years) ≤ 27 > 27	11 (16.2) 12 (17.6)	24 (35.3) 21 (30.9)	0.799
Gender Female Male	15 (21.7) 8 (11.6)	29 (42.0) 17 (24.6)	1.000
Location Maxilla Mandible	8 (11,6) 15 (21,7)	8 (11.6) 38 (55.1)	0.135
Lesion Tumor Cyst	21 2	27 19	0.006*
Lesion size Small (< 3 cm) Large (> 3 cm)	15 (23.4) 7 (10.9)	32 (50.0) 17 (15.6)	0.557
Recurrence Yes No	6 (8.7) 17 (24.6)	38 (55.1) 8 (11.6)	0.527
Odontogenic Tumors			
Histogenetic origin Epithelial Mixed Mesenchymal	4 (8.3) 10 (20.8) 7 (14.6)	14 (29.2) 12 (25.0) 1 (2.1)	0.008*
Benign/Malignant Benign Malignant	20 (45.5) 1 (25.0)	24 (54.4) 3 (75.0)	0.621
TOTAL	23 (33.3)	46 (66.7)	

*p ≤ 0.005

**Fig. 2:**
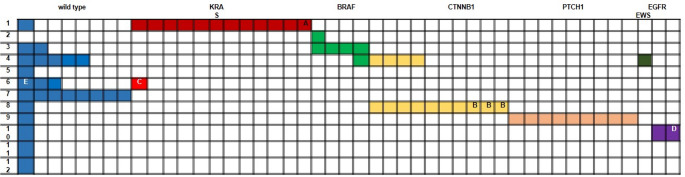
Mutational status across odontogenic lesions (*n* = 69). Lesions are coded as follows: 1= adenomatoid odontogenic tumor (AOT), 2 = odontogenic sarcoma (formerly ameloblastic fibrosarcoma), 3 = ameloblastic fibroma (AF), 4 = odontoma, 5 = dentinogenic ghost cell tumor, 6 = odontogenic fibroma, 7 = odontogenic myxoma, 8 = calcifying odontogenic cyst (COC), 9 = odontogenic keratocyst (OKC), 10 = clear cell odontogenic carcinoma (CCOC), 11 = calcifying epithelial odontogenic tumor (CEOT), 12 = squamous odontogenic tumor (SOT). Annotations: (**A**) Case with KRAS and additional KEAP1 mutation of uncertain significance. (**B**) Three cases with DDR2 mutations of uncertain significance and one case with activating KRAS mutation alongside CTNNB1 mutation. (**C**) KRAS and FGFR1 mutations detected; FGFR1 alteration is of uncertain significance. (**D**) EWSR1 translocation observed together with a TP53 mutation likely associated with loss of function. (**E**) One case with TP53 mutation not predicted to affect protein function (likely benign). *All mutations are classified according to the COSMIC database

**Fig. 3:**
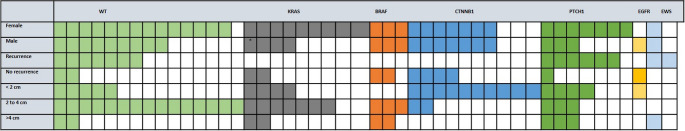
Association between mutation status and clinicopathological parameters in odontogenic lesions. Statistical correlations between genetic alterations and clinical variables such as age, gender, lesion type, and recurrence are shown. Note: One case exhibited concurrent KRAS and FGFR1 mutations (FGFR1 alteration of uncertain significance)

Of the 69 lesions successfully sequenced, somatic mutations were detected in 46 cases (66.7%), while 23 cases were wild-type (WT). The remaining 19 cases could not be sequenced due to degraded DNA or insufficient material (Figs. 2 and 3).

Multiple mutations were identified in 11 cases (24%), predominantly among cystic lesions such as COC and OKC (*p* = 0.001).

**Fig. 4:**
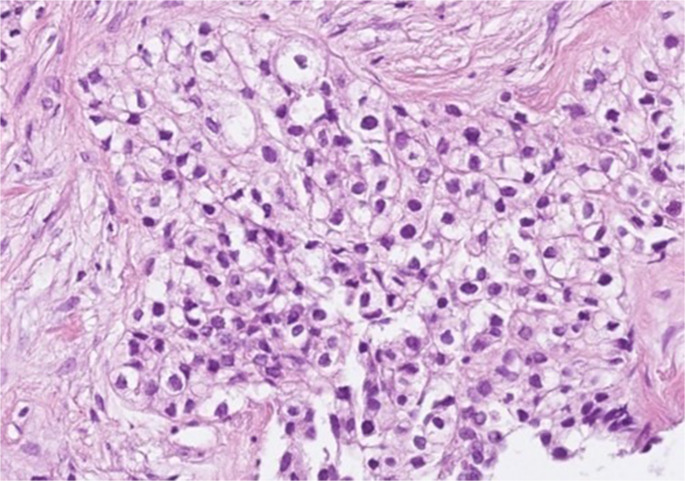
Representative case of odontogenic clear cell carcinoma (CCOC). Hematoxylin and eosin staining shows sheets and nests of clear cells (original magnification ×200). The patient died one year after initial diagnosis due to multiple pulmonary metastases

- The most frequent alterations involved KRAS and CTNNB1 genes:
  - KRAS p.G12V (c.35G > T) was detected exclusively in all sequenced AOT cases (*p* < 0.001).
  - CTNNB1 mutations were found in 66.7% of COC and odontoma cases.
  - PTCH1 mutations were present in 90% of OKCs, showing high statistical significance (*p* < 0.001).
- BRAF p.V600E mutations, classically linked to mandibular ameloblastomas, were also found in:
  - Four AF cases (66.6%).
  - One odontoma.
  - One odontogenic sarcoma.
- Rare mutations were detected in *EGFR*, *FGFR1*, and *TP53*—each in a single case.
- EWSR1 translocation was identified by FISH in two recurrent CCOC cases (*p* < 0.001), supporting its possible role in tumor progression (Fig. 4).

Mutation rates were higher in cystic lesions (76.0%) compared to tumors (47.6%) (*p* = 0.006). Epithelial odontogenic tumors had a significantly higher mutational burden than mixed or mesenchymal types (*p* = 0.008). Among malignant tumors, 75% harbored mutations (*p* = 0.05). In contrast, all odontogenic myxomas were wild-type (*p* < 0.001).

In the recurrent group (*n* = 7), only two CCOC cases showed detectable mutations. Four were WT, and one was excluded due to degraded DNA.

More than half of the odontoma cases (54.2%) were not suitable for sequencing, likely due to prior formic acid decalcification. Similarly, the single cases of SOT and CEOT could not be evaluated due to poor DNA quality. These findings highlight the critical need for optimized tissue processing protocols in molecular pathology of odontogenic lesions.

## Discussion

This study evaluated the mutational landscape of a diverse cohort of odontogenic lesions, excluding ameloblastomas which were comprehensively analyzed in our previous work. In that study, mandibular ameloblastomas predominantly harbored BRAF p.V600E mutations, while maxillary counterparts often carried SMO alterations [8, 15, 16]. In contrast, this investigation specifically included OKCs due to prior evidence of PTCH1 mutations in both sporadic and syndromic forms [5, 6]. These molecular findings lend further support to the concept of OKCs as neoplastic entities, previously recognized in the 2005 WHO classification as keratocystic odontogenic tumors.

The use of next-generation sequencing (NGS) in odontogenic pathology represents a significant advancement, offering insights into lesions with overlapping morphology but divergent biological behavior [7]. Odontogenic tumors originate from tooth-forming tissues and exhibit a broad clinical spectrum, from indolent growth to aggressive, recurrent, and even metastatic disease [16]. Histopathological diagnosis alone often lacks sufficient resolution, particularly in small biopsies or cystic lesions. NGS overcomes many of these challenges by enabling detection of somatic mutations and establishing genotype–phenotype correlations that inform both classification and therapeutic strategy [7, 14].

Our findings expand the known role of BRAF p.V600E mutations beyond ameloblastomas. In this study, the mutation was identified in 66.6% of AF cases, one odontoma, and one odontogenic sarcoma (previously ameloblastic fibrosarcoma). Traditionally, odontogenic sarcomas were thought to arise either de novo or through malignant transformation of recurrent AFs [17, 18]. The identification of BRAF mutations in these tumors provides strong evidence for a shared oncogenic driver across the benign–malignant spectrum of mixed odontogenic neoplasms. These findings not only support the concept of a molecular continuum linking AF and OS but also highlight the potential diagnostic value of BRAF mutation analysis, particularly in cases where morphology is ambiguous [16,18,19,20.]. Moreover, molecular testing is crucial in AF and OS to complement histopathology, supporting the potential diagnostic utility of BRAF mutation testing. in pleomorphic cases. Identifying mutation status may aid diagnosis and could, in selected cases, provide a rationale for exploring targeted therapeutic options supporting the notion that at least a subset of cases may exhibit neoplastic characteristics [18, 19].

Our results confirm the absence of BRAF p.V600E mutations in adenomatoid odontogenic tumors (AOTs), in agreement with their recognized distinction from ameloblastomas in current WHO classifications [2]. Instead, AOTs frequently harbor KRAS mutations, further supporting their unique molecular profile among benign odontogenic tumors [20–23].

We also identified TP53 mutations in one case each of odontogenic fibroma and clear CCOC. While TP53 alterations are rare in benign odontogenic tumors, their presence may indicate latent genomic instability or malignant potential under certain conditions. In CCOC, TP53 mutation aligns with its known aggressive clinical behavior and supports a role for TP53 as a driver of tumorigenesis and progression [3, 9].

Additionally, EWSR1 translocations, commonly associated with Ewing sarcoma and related tumors, were found in two recurrent CCOC cases. This novel observation, statistically significant (*p* ≤ 0.001), suggests that EWSR1 rearrangements may serve as a molecular marker of recurrence in CCOC. The identification of EWSR1 rearrangements in CCOC represents an important advance in refining the diagnosis of this rare odontogenic malignancy [8, 15, 24]. Traditionally, CCOC has posed diagnostic challenges due to its histopathological overlap with other clear cell–rich odontogenic and salivary gland tumors [25]. The demonstration of EWSR1 translocations in a subset of CCOCs establishes a molecular link with hyalinizing clear cell carcinoma (HCCC) of the salivary glands, which is also characterized by EWSR1 rearrangements. This shared genetic alteration raises the possibility that EWSR1-rearranged CCOCs may represent a central counterpart of HCCC, particularly in cases showing intraosseous involvement with extension to adjacent soft tissues [24, 25]. While the current evidence suggests that EWSR1 rearrangements in CCOC primarily serve as a diagnostic marker rather than a prognostic factor, their detection by FISH or molecular analysis can greatly aid in distinguishing CCOC from histologic mimics such as clear cell calcifying epithelial odontogenic tumor CEOT [26]. Furthermore, as molecular profiling may become more integrated into routine diagnostic practice, the recognition of this translocation not only strengthens the biological relationship between odontogenic and salivary clear cell carcinomas but may also open avenues for future targeted therapies directed at translocation-driven oncogenesis in recurrent or aggressive cases.

A key observation in our study was the high frequency of PTCH1 mutations in OKC cases, which likely reflects both the neoplastic nature of these lesions and the increased sensitivity of DNA extraction through microdissection. Although the WHO reverted OKC to a cystic entity in 2017 and 2022 due to lack of consensus, our findings add to a growing body of literature advocating for its reconsideration as a neoplasm in at least a subset of cases [2, 5–7, 9].

An interesting and somewhat unexpected finding of the present study was the observation that cystic lesions demonstrated a higher mutation rate (76%) compared to tumors (47.6%). This result may, at first glance, appear counterintuitive, as neoplastic tumors are generally assumed to harbor more frequent genetic alterations than cystic lesions [1–4]. However, several factors may explain this discrepancy. First, the study design selectively excluded non-neoplastic cysts, such as dentigerous cysts, from the analysis. This exclusion likely enriched the cystic cohort with lesions that have a well-established neoplastic potential, including odontogenic keratocysts and calcifying odontogenic cysts, which are known to harbor recurrent mutations in oncogenic pathways. Indeed, aberrations in the MAPK, Sonic Hedgehog (SHH), and WNT/β-catenin pathways are well documented in odontogenic cysts and tumors, with BRAF, PTCH1, and CTNNB1 representing the most frequently altered genes [7–12]. In contrast, the tumor cohort in this study excluded ameloblastomas, which are characterized by a particularly high prevalence of BRAF mutations [7, 8, 12, 13]. Since ameloblastomas constitute the most mutation-rich odontogenic tumors, their omission from the tumor group likely resulted in an underestimation of mutation frequency among tumors overall.

In the present cohort, all odontogenic myxomas (OMs) were wild-type (*p* < 0.001), consistent with the findings of most previous studies reporting the absence of recurrent driver mutations in these tumours [27, 28]. While PRKAR1A, PIK3CA (Q546E) and KRAS mutation (G12V), mutations have been detected in a minority of OMs [29, 30] and GNAS1 mutations are well described in intramuscular myxomas, neither has emerged as a consistent molecular feature of OMs. Furthermore, targeted and broad next-generation sequencing analyses have not revealed pathogenic mutations in genes commonly altered in human cancers, although isolated findings such as copy number variations and chromosomal abnormalities have been reported in select cases [31]. These observations suggest that OMs are molecularly distinct from other odontogenic neoplasms and may rely more on epigenetic dysregulation, as demonstrated by hypomethylation of tumor suppressor genes including CDKN1A, CDKN1B, CDKN2A, TP53, and RB1 [31, 32]. The results of this study lend support to the hypothesis that the molecular pathogenesis of OMs remains poorly understood, with current evidence indicating a heterogeneous and likely epigenetic basis rather than recurrent oncogenic mutations.

The utility of molecular profiling is particularly evident in limited biopsy samples, where classic histology may be inconclusive. In this context, genotyping can assist in distinguishing between overlapping entities and guiding surgical decisions. Although no clear prognostic patterns were identified—largely due to incomplete clinical follow-up data—future studies correlating mutational status with clinical outcomes are warranted.

From a therapeutic standpoint, certain mutations may represent potential predictive biomarkers, although this requires further validation. Preliminary clinical experience with BRAF inhibitors, including our group’s work, either as a monotherapy or in combination with MEK inhibitors, has already yielded encouraging results in ameloblastomas [15, 33, 34]. This suggests that analogous strategies could be advantageous for patients with other BRAF-positive odontogenic neoplasms. Beyond the reduction of tumour burden and recurrence, neoadjuvant BRAF/MEK inhibition has the potential to facilitate organ-preserving surgeries, minimise functional and aesthetic impairment, and enhance long-term quality of life [35, 36].

Furthermore, the potential of SMO and PTCH1 mutations to guide the treatment of selected odontogenic tumours warrants further investigation [19, 37, 38].

## Limitations

This study has several limitations. First, DNA degradation due to decalcification and long-term storage led to the exclusion of 19 cases, which may have influenced mutation detection rates. Second, some rare odontogenic tumor subtypes were represented by only one or two cases, limiting the strength of statistical comparisons. Third, the cohort was restricted to referral centers in Turkey and Germany, which may introduce geographic or population-related bias. Finally, survival and long-term clinical outcome data were not consistently available, precluding robust prognostic correlations. Future multi-institutional studies with larger and more diverse cohorts, optimized tissue processing, and comprehensive follow-up data will be essential to validate and extend our findings.

Although next-generation sequencing (NGS) provides valuable molecular insights into odontogenic lesions, its routine implementation in diagnostic pathology faces several challenges. High costs, limited availability of sequencing infrastructure, and the need for specialized bioinformatics support restrict widespread use, particularly in low-resource settings [38].

This study is limited by the relatively small sample size of certain histological subtypes and the retrospective design. Additionally, the NGS panel used covered only a subset of cancer-related genes, and therefore some relevant alterations might have been missed. Nevertheless, our findings should be interpreted within the context of the limited sample size and targeted gene panel used, and warrant confirmation in larger, multi-institutional cohorts.

## Data Availability

The datasets generated and/or analyzed during the current study are not publicly available due to ethical restrictions but are available from the corresponding author on reasonable request.
